# Supplementary material for: Protocol for a Randomized Controlled Trial to Enhance Executive Function via Brief Mindfulness Training in Individuals with Internet Gaming Disorder
Source: PLoS One. 2025 Apr 1;20(4):e0320305. doi: 10.1371/journal.pone.0320305 (PMC11960939; doi:10.1371/journal.pone.0320305)
Supplement: S1 File — (DOCX) [file pone.0320305.s001.docx]

**S1 File. Copy of the study protocol approved by the ethics committee**

**The effect of brief mindfulness intervention on executive function in individuals with Internet Gaming Disorder**

1. ***Declaration of Integrity. [including two aspects: (1) guaranteeing that the operation strictly follows the experimental procedures and the authenticity of data records; (2) truthfully disclosing any conflicts of interest]***

The research will ensure that all operations are strictly follow the trial procedures and authenticity of data records; There are no interests conflicts in this study.

1. ***Title. [The research title should accurately reflect the research purpose, be concise and brief, and adhere to the PICOS structure, which includes the disease or research subject, treatment methods, control methods, outcomes or research objectives, and research or design type. （participants, intervention, control, outcome, study）]***

The effect of brief mindfulness intervention on executive function in individuals with Internet Gaming Disorder

1. ***Protocol version (Date and version identifier)***

V1.0

1. ***Funding (Sources and types of financial, material, and other support)***

Yunnan Fundamental Research Projects (202401AT070332)

1. ***Flowchart or table of the research implementation process, with SPIRIT template attached. [arranging tasks to be performed at each time point of the study].***


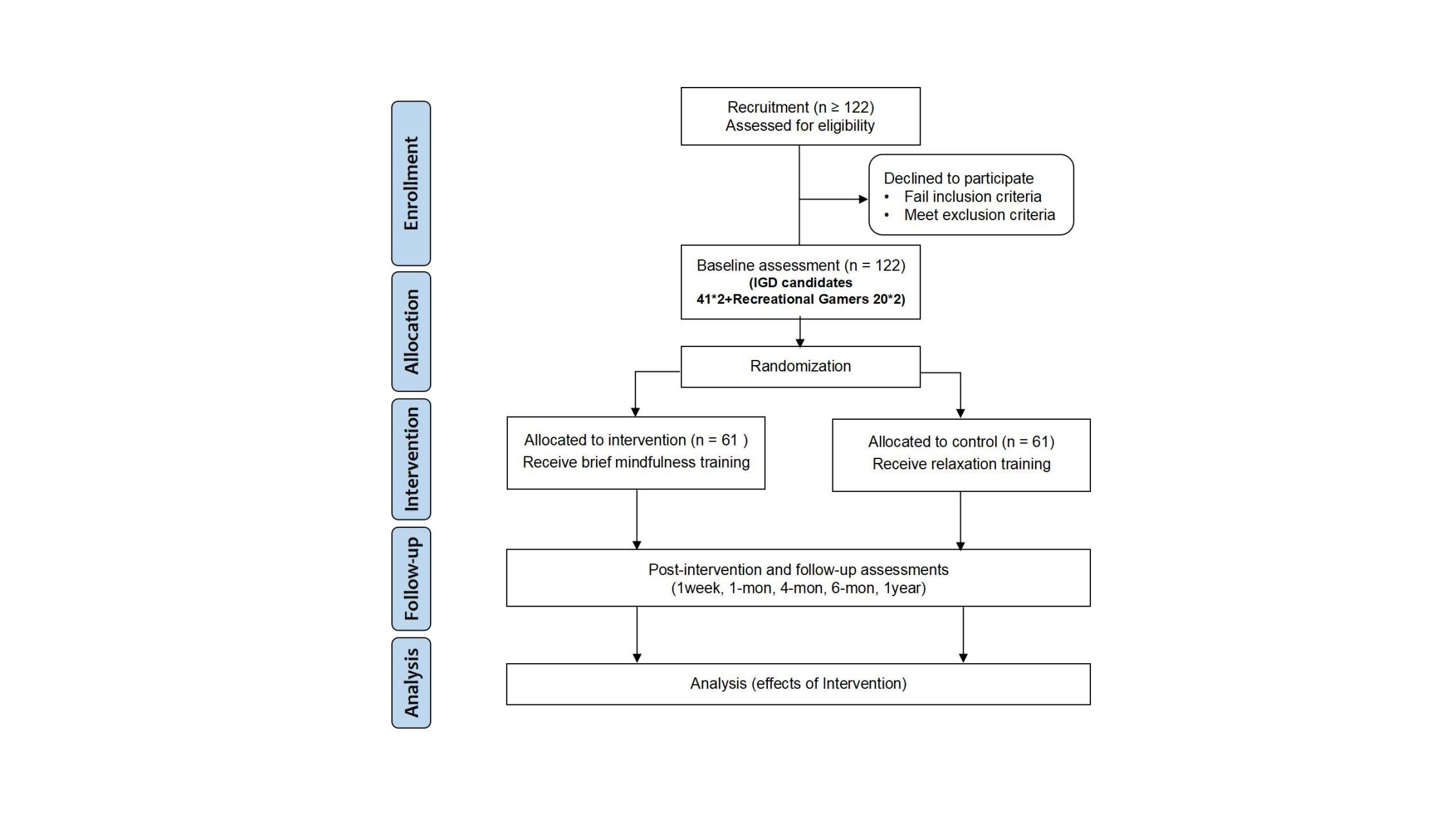


**Fig 1. SPIRIT Flowchart**

|  | **STUDY PERIOD** | | | | | | | |
| --- | --- | --- | --- | --- | --- | --- | --- | --- |
|  | **Enrolment** | **Allocation** | **Post-allocation** | | | | | **Close-out** |
| **TIMEPOINT*** | ***-t_2_*** | ***-t_1_*** | ***t_0_*** | ***t_1_*** | ***t_2_*** | ***t_3_*** | ***t_4_*** | ***t_5_*** |
| **ENROLMENT:** |  |  |  |  |  |  |  |  |
| **Eligibility screen** | X |  |  |  |  |  |  |  |
| **Informed consent** | X |  |  |  |  |  |  |  |
| **Baseline assessment** | X | X |  |  |  |  |  |  |
| **Allocation** |  | X |  |  |  |  |  |  |
| **INTERVENTIONS:** |  |  |  |  |  |  |  |  |
| ***[Brief mindfulness training]*** |  |  |  |  |  |  |  |  |
| ***[Relaxation training]*** |  |  |  |  |  |  |  |  |
| **ASSESSMENTS:** |  |  |  |  |  |  |  |  |
| ***[Demographic data]*** | X |  |  |  |  |  |  |  |
| ***[Primary outcome variables]*** | X | X | X | X | X | X | X | X |
| ***[Secondary outcome variables]*** | X | X | X | X | X | X | X | X |
| ***[Daily records during intervention]*** |  |  | X | X | X | X | X | X |

**Fig 2. SPIRIT schedule of enrolment, interventions and assessment.**

** Note: -t2, -t1, baseline (complete measurements twice to get average); t0, post-intervention; t1- t5, 1-week, 1-month, 4-month, 6-month,1-year follow-up.*

1. ***Research background. [usually adopting a four-part structure: (1) Overview of the health problem or research question: definition of the health problem, epidemiological data, etiology, natural history of the disease, disease burden, etc.; (2) Scientific hypothesis: mechanism or principle; (3) Clinical application, for example, treatment studies should explain the treatment methods; (4) Necessity of the study based on current evidence, explaining why this study needs to be conducted]***

According to the latest data from the China Internet Network Information Center [[1](#_ENREF_1)], by June 2022, the scale of online game users in our country still exceeded half of the total internet user base, reaching 552 million, among whom mobile games dominated, accounting for 60.9% of underage gamers [[2](#_ENREF_2)]. Unlike substance addiction, which is induced by addictive substances, internet gaming disorder (IGD) is considered a behavioral addiction, characterized prominently by uncontrollable gaming behavior leading to emotional distress, neglect of academic or life responsibilities, and damage to interpersonal relationships, causing serious negative impacts on both individuals and society [[3](#_ENREF_3)]. According to the dual-process model, cognitive-behavioral model, and I-PACE model in addiction theory, it is concluded that emotional and cognitive mechanisms jointly contribute to the development and maintenance of gaming addiction, with cue-induced craving and diminished executive function closely related to IGD. Executive function regulation is also required for cue-induced craving, making executive function a critical area of research in IGD. The primary behavioral diagnostic feature of IGD is excessive gaming behavior despite the negative consequences, and behind this behavioral dysfunction lies functional impairment. Studies using fMRI on individuals with IGD have found abnormalities in brain structures such as the dorsolateral prefrontal cortex, anterior cingulate cortex, and orbitofrontal cortex, areas closely associated with executive function. However, studies on the executive function of IGD individuals have mainly focused on the subcomponent of inhibitory control, with less attention given to switching and updating components, particularly regarding the cognitive flexibility represented by switching, which remains controversial in the field of internet gaming addiction. To better explore the neurobiological mechanisms related to internet gaming addiction, it is necessary to investigate all three subcomponents of executive function.

Mindfulness, as an effective addiction treatment approach, has been widely applied in interventions for addiction and relapse, improving craving and maladaptive cognition in individuals with IGD [[4](#_ENREF_4)], and has been proven to significantly enhance prefrontal cortex function, which is closely related to executive function. Individuals can improve overall executive function by initiating attention monitoring processes, strengthening working memory, and increasing cognitive flexibility in task switching, thus reducing impulsivity and addictive behaviors. Research on mindfulness interventions concerning executive function has primarily targeted populations such as patients with brain injuries, children with underdeveloped brain mechanisms, and elderly individuals with declining brain functions; there is less research on normally developed, healthy adults, especially within the addicted population, where the focus has largely been on smokers rather than individuals with IGD. Moreover, due to the time-consuming nature and face-to-face requirements of mindfulness interventions, the demand for short-term self-guided practices has increased. Existing research has confirmed that single-session and five-minute brief mindfulness interventions can improve health outcomes.

Currently, whether the three subcomponents of executive function in individuals with IGD are impaired, and whether mindfulness interventions can improve executive function and related brain mechanisms in IGD populations and normal healthy populations, lacks sufficient in-depth investigation. This study proposes to use a randomized controlled trial design to explore the effects of a one-week focused breathing meditation practice on executive function. On one hand, this may address the issue of low acceptance due to the long duration of mindfulness interventions, and on the other hand, exploring brain function mechanisms could provide more targeted intervention strategies for executive function research.

1. ***Objective***

Through the use of scales, behavioral data, and EEG data, this study aims to investigate whether the three sub-components of executive function in individuals with internet gaming addiction are impaired. Furthermore, it will conduct a one-week focused breathing meditation intervention to explore the effectiveness of concise mindfulness on executive function and its impact on brain function mechanisms. This endeavor will provide more targeted intervention strategies for research into executive function.

1. ***Inclusion and exclusion criteria. [Inclusion criteria define the main body of the study and determine the representativeness of the study population; exclusion criteria define individuals within the study population that may affect the study and determine the homogeneity of the study population]***

Inclusion criteria:

(1) Right-handedness;

(2) Normal vision or corrected-to-normal vision;

(3) Age between 18 and 40 years;

(4) Good mental and physical health (PHQ-9 total score < 20, GAD-7 total score < 11);

(5) Absence of other addictions (e.g., smoking, alcohol, gambling);

(6) For individuals with internet gaming disorder (IGD): Internet Addiction Test (IAT) score ≥ 50, DSM-5 score ≥ 5, gaming history exceeding 2 years, and gaming time of more than 14 hours per week;

(7) For recreational gamers: IAT score < 50, DSM-5 score < 5.

Exclusion criteria:

(1) Individuals with asthma, epilepsy, or a history of mental illness;

(2) Those who have taken steroid medications within the past three months;

(3) Individuals who have practiced meditation, yoga, Tai Chi, or Qi Gong for more than 20 hours in the past year or throughout their lifetime, attended meditation or yoga retreats, or participated in any meditation courses;

(4) Individuals unsuitable for electroencephalogram (EEG) testing due to metal implants, severe head injuries, contact dermatitis, or silicone allergies;

(5) Those with a PHQ-9 total score ≥ 20 or a GAD-7 total score ≥ 11;

(6) Individuals with other addictions (such as smoking, alcohol, or gambling);

(7) Individuals with strong adherence to a particular religious belief that would interfere with their ability to participate in the required meditation exercises;

(8) Individuals currently participating in similar trials or other neurophysiological studies.

1. ***Study design, design pattern diagram, with SPIRIT template attached. [Clearly state the design scheme used in this study, such as: consecutive case or single-arm design, cross-sectional study, case-control study, cohort study, including historical cohort study or prospective cohort study, nested case-control study, randomized controlled trial, clearly specify the type, such as parallel design, crossover design, factorial design, single-group design; for treatment studies, specify the type of research objective framework, such as superiority, equivalence, non-inferiority, exploratory]***

The current trial adopts a randomized controlled parallel design, with substantial literature supporting the moderate effect of brief mindfulness interventions on executive function [[5](#_ENREF_5)]. Therefore, for the IGD population, this trial aims to demonstrate superiority under the condition of a brief mindfulness intervention compared to the control group. The trial comprises four stages: participant recruitment, baseline assessment, post-intervention measurement, and follow-up assessment, with corresponding evaluations conducted at the conclusion of each phase. A schematic diagram of the design is depicted in **Fig 3.** The trial has not yet commenced, and it is estimated that the entire process from initiating participant recruitment to completing data analysis and manuscript writing will take approximately one year.

**
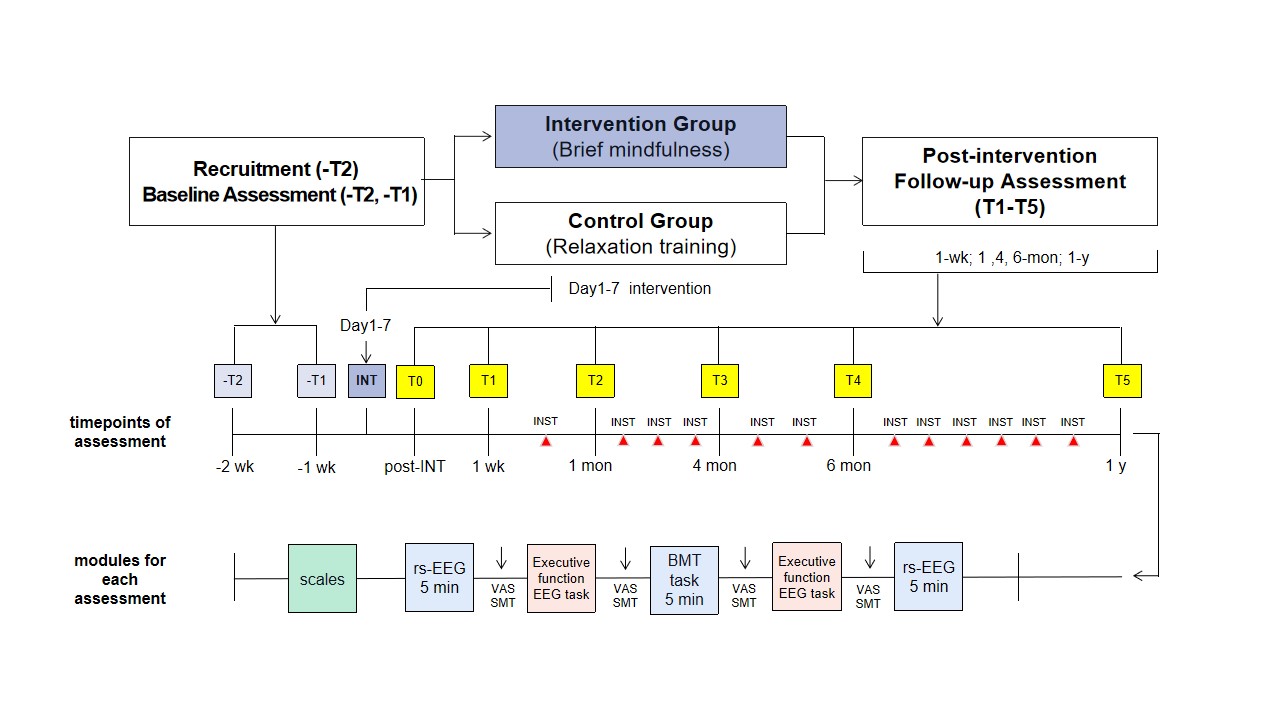
**

**Fig 3. Study Design and Procedure**

*The upper portion indicates the overall study design. The middle portion indicates the timeline for assessment of scales and EEG at baseline and post-intervention, also follow-up time-points. Brief mindfulness and relaxation training was administered to intervention group and control group after recruitment.Baseline assessment (-T2, -T1) was finished before intervention. Audio guidance was delivered for everyday's practice for BMT group. There is no other practice and intervention for control group after training. The lower portion indicates modules of psychological and behavioral assessments during the baseline and 5 follow-up sessions. Executive function measurement was implemented via EEG tasks including Stroop, More-odd Shifting, N-back. INT: intervention, INST:monthly intensive training (red triangle); rs-EEG: resting-state EEG; BMT: brief mindfulness training; VAS: Visual analogue scale; SMT: state-mindfulness.*

1. ***Sample size estimation. [List the four elements: δ(Δ) or π1, π2, α, β or Power, and provide the calculation formula]***

In this trial, Stroop reaction time related to executive function will be used as the primary indicator, and the sample size estimation will be conducted using the pwr package in R software. It is set that δ(Δ)tot = 125, corresponding to an expected reduction of 125ms in reaction time for the experimental group after the intervention. Additionally, α is defined as 0.05 and β as 0.80.

The estimation of Cohen's d is required, with the formula Cohen's d = (δ(Δ) / SDestimate). Referencing studies in similar populations [<https://www.ncbi.nlm.nih.gov/pmc/articles/PMC8939409/>], SDestimate is taken as 250. Therefore, the relevant command is pwr.t.test (d = cohen_d, sig.level = 0.05, power = 0.80, type = "paired", alternative = "two.sided"), resulting in a sample size estimate of 34. Thus, the estimated sample size for the experimental group should be no less than 34 individuals; considering a natural dropout rate of 20%, the sample size for the experimental group should be at least 41 individuals. A ratio of 2:1 will be used to include 20 recreational gamers in the intervention group. A total of 61 participants are required for the intervention group. In order to maintain appropriate comparability, we will recruit an additional 61 participants with matching demographic profiles and equivalent levels of IGD (Internet Gaming Disorder) severity for the control group. Thus, the study demands a cumulative participation of 122 individuals in total.

1. ***Methods of randomization and concealed allocation. [Randomization is used to reduce the risk of selection bias. Methods for generating random number sequences include computer software, random number tables, semi-random or pseudo-random methods (such as using dates, birthdays, or even/odd numbers of hospital visits). Please describe the individuals involved in generating random number sequences and the methods they use. Concealed allocation is used in treatment studies to reduce the risk of selection bias. Key steps in concealed allocation include having a designated person determine the allocation sequence, ensuring that this person is not involved in enrolling participants, and securely storing the allocation table. Please provide detailed descriptions of these steps. Central randomization is a robust method of concealed allocation. Describe the individuals and methods involved in determining the allocation sequence]***

(1) In this study, the intervention period focused on breath meditation lasts for one week and serves as a concise mindfulness behavior training. The study will involve a baseline collection of EEG and other relevant data for all participants, a one-week training on the intervention content, collection of post-intervention EEG and other relevant data, and follow-ups (one week, one month, four months, six months, and one year after data collection post-intervention). During the follow-up phase, participants will undergo multiple mindfulness training sessions. Grouping will occur after the baseline data collection for participants is completed.

(2) The breath meditation training is the primary intervention method employed in this study. The design includes grouping into an experimental group and a control group: the experimental group (breath meditation training) and the control group (relaxation training). The recruited participants are from the same school, and their connections make it challenging to implement the criteria for double-blind randomization.

(3) The randomization process for this trial will utilize the random sequence generator available at random.org.

1. ***Blinding. [Blinding is used in treatment studies and diagnostic trials to reduce the risk of implementation bias and measurement bias. Implementing strict confidentiality measures, such as using placebos or simulating procedures, is an effective blinding method. If blinding is necessary and feasible for your study, describe the methods and procedures for implementing blinding]***

This trial involves group therapy, with participants being students from the same school. Therefore, it is possible that among participants who may know each other or even among those who do not, conversations related to the experimental training they are undergoing may arise. Additionally, maintaining blinding for the researchers is quite challenging, as they can easily be influenced by information related to the experiment during the process. As a result, it is not feasible to meet the double-blind requirements of clinical trials.

1. ***Outcome Measures. [Outcome measures are used to evaluate the results of a study and can include primary, secondary, and safety measures, including adverse events and reactions. Please describe the names of the measures, the methods of measurement, and the time points at which they are assessed]***

(1) Primary indicators: average daily gaming time (self-reported by participants), Five Facet Mindfulness Questionnaire (FFMQ), Internet Addiction Test (IAT), Game Craving Scale (adapted from QSU-Brief), Adolescent Executive Functioning Scale (EFS-A), Barratt Impulsiveness Scale, State Mindfulness Scale, Game Craving Visual Analogue Scale, behavioral data, and EEG data (from EEG equipment).

(2) Secondary indicators: Self-Rated Sleep Status Scale, Depression-Anxiety-Stress Scale (DASS-21), Positive and Negative Affect Schedule (PANAS).

(3) Safety indicators: Researchers will inquire daily during the intervention period whether participants experience any adverse reactions. If there are any adverse reactions, they will communicate in person or by phone with the research staff.

Measurement time points are detailed in item 5.

1. ***Definition of Participant Effectiveness Determination. [This includes definitions for participant withdrawal, exclusion, loss to follow-up, confounding factors, study discontinuation, and study suspension]***

(1) Participant Withdrawal: Each participant can withdraw from the study at any time during the experiment without providing any reason. Participants who withdraw voluntarily will not be included in the analysis. Under the conditions of a mixed study, we have a complete access procedure for withdrawn participants. If participants are willing to provide a reason for their withdrawal, it will be recorded by the researchers.

(2) Participant Exclusion: If participants cannot consistently participate in the group interventions during the training process—where the focused breathing meditation training lasts one week—those who attend fewer than 5 sessions will be considered not fully intervened and should theoretically be excluded or adjusted using a mixed-effects model.

(3) Participant Loss to Follow-up: If any participant cannot be contacted for two consecutive attempts after the intervention is completed, they will be classified as lost to follow-up.

(4) Participant Contamination: After the intervention is completed, we will conduct a census of participants. If any participant in the control group attempts to learn about the training methods of the intervention group (or vice versa) and tries to train, they will be classified as contaminated participants.

(5) Participant Termination: To ensure the health of the participants, any adverse events will constitute a termination condition. Researchers will discuss such situations with the affected participants to consider premature termination of their intervention. Appropriate clinical interventions will be provided to them with their consent.

(6) Participant Suspension: In this trial, we will allow participants to request suspension of their involvement in the intervention. If any requests for suspension are made, participants will be moved to a future intervention group after the trial team assesses that no adverse events or reactions have occurred. Data collected from these participants during the current trial will not be included in the final data analysis.

| **Table 1. Evaluated variables**   \| **Variables** \| \| **Measures** \| **Timepoints** \| \| --- \| --- \| --- \| --- \| \| **Primary outcome** \| **Trait-, State-mindfulness** \| Five Facet Mindfulness Questionnaire (FFMQ) [[6](#_ENREF_6)] \| -T2, -T1; T0, T1, T2, T3, T4, T5 \| \| State Mindfulness Scale [[7](#_ENREF_7)] \| \| **Electroencephalographic (EEG) and behavioral data** \| Behavioral and Electroencephalogram (EEG) data \| \| Heart Rate Variability \| \| **Gaming craving-related metrics** \| Average daily gaming time (self-recorded by participant) \| \| Internet Addiction Test (IAT) [[8](#_ENREF_8), [9](#_ENREF_9)] \| \| Gaming Craving Visual Analogue Scale [[10](#_ENREF_10)] \| \| Gaming Craving Short Questionnaire (adapted from QSU-Brief) [[11](#_ENREF_11)] \| \| **Impulsivity** \| Barratt Impulsiveness Scale [[12](#_ENREF_12)] \| \| **Executive function** \| Adolescent Executive Functioning Scale (EFS-A) [[13](#_ENREF_13)] \| \| **Secondary outcome** \| **Sleep status** \| Self-Rating Scale of Sleep (SRSS)[[14](#_ENREF_14)] \| -T2, -T1; T0, T1, T2, T3, T4, T5 \| \| **Affect, Stress** \| Depression Anxiety Stress Scales (DASS-21) [[15](#_ENREF_15)] \| \| Positive and Negative Affect Schedule (PANAS) [[16](#_ENREF_16)] \| \| **others** \| **Socio-demographic and clinical information** \| Demographics including name, gender, handedness [[17](#_ENREF_17)], gaming history duration, average weekly gaming time, psychiatric diagnosis or history of mental health problems \| -T1 \| \| **Psychological status** \| Patient Health Questionnaire-9 (PHQ-9) [[18](#_ENREF_18)] \| \| Generalized Anxiety Disorder-7 (GAD-7) [[19](#_ENREF_19), [20](#_ENREF_20)] \| \| **Presence of IGD** \| Diagnostic and Statistical Manual of Mental Disorders, Fifth Edition (DSM-5) nine diagnostic criteria [[21](#_ENREF_21)] \| \| Gaming history, average weekly gaming time \| \| **Personal traits** \| Chinese Big Five Personality Questionnaire [[22](#_ENREF_22)], attachment style [[23](#_ENREF_23)], self-compassion [[24](#_ENREF_24)] \| |
| --- | --- | --- | --- | --- | --- | --- | --- | --- | --- | --- | --- | --- | --- | --- | --- | --- | --- | --- | --- | --- | --- | --- | --- | --- | --- | --- | --- | --- | --- | --- | --- | --- | --- | --- | --- | --- | --- | --- | --- | --- |

1. ***Definition, identification methods, and management systems for adverse events and reactions.***

Dr. Farias [[25](#_ENREF_25)] systematically describes the adverse events and reactions caused by meditation (meditation adverse events, MAEs) in the text, which we categorize as follows:

**Mental MAEs:** The most common include anxiety and depression, while other relatively common issues include psychotic or delusional symptoms, dissociation or depersonalization, fear or phobia, trauma re-experiencing, and suicidal thoughts and behaviors.

**Physical MAEs:** The most common is bodily tension, followed by pain and gastrointestinal issues. Localized pain often involves the abdomen/stomach and neck. Most physical MAEs are based on patient self-reports; however, some variables also used physiological and biological measurement methods, such as heart rate, blood pressure, skin conductance, and cortisol levels (it is not possible to rule out that these physiological measuring methods might cause side effects).

**Neurological/Cognitive MAEs:** The most common are abnormal cognitive experiences, including thought disorganization, forgetfulness, sensory hypersensitivity, and impaired memory reliability. Some patients also experienced involuntary bodily movements and muscle contractions during meditation.

Based on the types of adverse effects mentioned above, to address adverse events during the intervention process, we have included relevant adverse reaction feedback questions in the weekly follow-up questionnaires. The experimental group will conduct weekly monitoring; after collecting the questionnaires, if an adverse event feedback option is marked "yes," the relevant person in charge from the medical school and psychology department will be responsible for communicating with the subjects, assessing their psychological and physiological conditions, and gauging the subjects' willingness. After a tripartite consensus (medical school, psychology department, and the individual subject), they will decide whether the subjects experiencing adverse events can participate in subsequent research.

Secondly, regarding adverse reactions during the EEG data collection process, if participants show physiological rejection responses or adverse emotions, researchers will immediately cease EEG data collection. Whether to continue the experiment will depend on ensuring the participants' health and safety, as well as their own willingness to either continue participating in the experiment or withdraw, with researchers recording the reasons. Subsequent follow-ups and interventions in this study will be communicated to participants after collective discussion among the research team before any decisions are made.

1. ***Ethical considerations. [This includes the selection of an ethics committee, approval procedures, informed consent process, registration time, and registration authority (please provide registration number after completion)]***

(1) Selection of the Ethics Committee: This trial has selected the ethics committee of the implementing institution, namely the Medical Ethics Committee of Kunming University of Science and Technology.

(2) Approval Process: Before the study begins, the research project proposal and related materials have been submitted for approval in accordance with the procedural requirements of the selected ethics review committee of the research institution.

(3) Informed Consent Process: Before the study begins, researchers will provide paper copies of the informed consent form to the participants and authorized agents, and address any questions related to the informed consent form to ensure that participants and authorized agents understand the content before signing the informed consent form.

(4) Registration Time: March 2024.

(5) Registration Institution: China Clinical Trial Registration Center.

1. ***Participant recruitment. [This includes the selection of recruitment locations, recruitment methods, the screening process, and the researchers conducting the screening]***
2. Recruitment Location: Chenggong Campus, Kunming Medical University.
3. Recruitment Method: This trial will use a combination of online and offline methods to recruit participants:

Offline: Conduct presentations in classrooms, create posters, and distribute flyers;

Online: Use campus platforms such as QQ Space, WeChat Moments, and the school's community bulletin board.

1. Screening Process: Participants will be selected based on their order of participation according to inclusion and exclusion criteria.
2. Researchers Implementing Screening: Screening will be conducted by specifically trained personnel assigned to the project.
3. ***Collection of general participant information. [This includes the researchers responsible for data collection and the content of the general participant information]***

(1) Researchers Collecting Data: The specialized personnel responsible for the aforementioned participant screening.

(2) Content of General Information: Name, gender, age, grade, major, ethnicity, dominant hand, years of gaming experience, average weekly gaming duration, whether currently participating in similar trials, physical and mental health status, and other relevant information.

1. ***Baseline measures and observed variables. [For treatment studies, observed variables may not necessarily be efficacy evaluation measures, such as height and weight, but may be related to drug dosage without direct relevance to efficacy evaluation]***

(1) Baseline Indicators: Demographic information (name, student ID, gender, age, major, ethnicity, contact information, dominant hand, history of mental illness or psychiatric history, PHQ-9, GAD-7, duration of gaming, average weekly gaming time), Five Facet Mindfulness Questionnaire (FFMQ), Internet Addiction Test (IAT), DSM-5 nine diagnostic criteria, Gaming Craving Short Questionnaire, Adolescent Executive Functioning Scale (EFS-A), Barratt Impulsiveness Scale, State Mindfulness Scale, Gaming Craving Visual Analog Scale, Sleep Condition Self-Assessment Scale, behavioral data and EEG data (electroencephalography device), Depression-Anxiety-Stress Scale (DASS-21), Positive and Negative Affect Schedule (PANAS).

(2) Observation Items: Average daily gaming time, Five Facet Mindfulness Questionnaire (FFMQ), Internet Addiction Test (IAT), Gaming Craving Short Questionnaire, Adolescent Executive Functioning Scale (EFS-A), Barratt Impulsiveness Scale, State Mindfulness Scale, Gaming Craving Visual Analog Scale, Sleep Condition Self-Assessment Scale, behavioral data and EEG data (electroencephalography device), Depression-Anxiety-Stress Scale (DASS-21), Positive and Negative Affect Schedule (PANAS). The measurement tools and specific content are shown in **Table 1**. The outcome level and timepoints for assessment was annotated.

1. ***Standard operating procedures. [For treatment procedures, treatment methods, treatment courses, etc.; diagnostic trial standard procedures; etiological research standard procedures; prognosis research standard procedures; epidemiological research standard procedures]***

Trained personnel rigorously adhere to the main operating procedures related to this research process. The standard operating procedures are as follows:

(1) **Procedure for EEG data collection:**

1. Pre-collection Preparation: Inform participants of relevant precautions and prepare for the collection (including both software and hardware; software refers to the specialized computer software that links to the EEG collection device, while hardware includes the EEG cap, conductive gel, and other necessary auxiliary equipment).
2. Measure Head Circumference: Measure the participant’s head circumference to determine the appropriate size of the EEG cap (Large cap: 56-60 cm; Small cap: 54-58 cm).
3. Prepare Long Hair: If the participant has long hair, it should be tied back with a hairband before putting on the EEG cap to expose the ears (participants can prepare the hairband themselves, or it will be provided by the laboratory).
4. Correct Placement of the Cap: Place the cap on the participant’s head correctly according to the front and back of the cap and the electrode positions (the side with letters facing up, with the letter F indicating the front and O indicating the back).
5. Positioning the Cz Electrode: Measure the distance from the nasal root to the inion, and adjust the EEG cap forward and backward so that the Cz electrode is in the center of this distance. Measure the distance between the left and right ear lobes, and adjust the cap left and right to make sure the Cz electrode is at the center of this distance. The Cz electrode should be at the intersection point of the two measured distances.
6. Visual Check: Conduct a visual inspection from the front of the participant to ensure the EEG cap does not rotate or become loose. If there is looseness or incorrect electrode positioning, repeat step “e)” until the EEG cap is appropriately positioned.
7. Applying Conductive Gel: Use a specialized syringe (angled syringe) to apply conductive gel to the scalp at the corresponding channels. Since the syringe is used to part the hair, ask the participant if the pressure applied is too forceful during the process and adjust accordingly based on their feedback.
8. Insert Active Electrodes: Correspondingly insert the labeled active electrodes into the designated electrode names marked on the EEG cap (make sure to carefully match the electrodes’ labels with the EEG cap). Wrap the CMS and DRL active electrode wires around the other electrodes' cables (3-5 wraps), and plug their connectors into the corresponding matching ports on the amplifier.
9. Confirm Connections and Start Data Collection: After confirming that the EEG software and hardware are properly connected, run the software to collect EEG data. Follow the software usage instructions and ensure the quality of the EEG data meets the required standards before starting the data collection. Save the EEG data to the computer or hard drive according to your naming format.
10. Intervention Content Training Process:

The researchers of this study will collaborate with professional psychological teachers to conduct a week-long intervention content training and multiple training sessions during the follow-up phase.Before each practice session begins, participants will be reminded of the time and location of the practice, and the researchers will conduct on-site check-ins. Training for the intervention content will be provided for one week by the teachers from the Student Mental Health Education and Counseling Center, along with multiple training sessions during the follow-up phase. Professional psychological teachers will guide the training, and the content practiced each day will be sent to the participants' group after each session for continued practice.

1. ***Statistical analysis methods. [Select appropriate statistical analysis methods based on the data type of each measurement variable. Include any additional methods for subgroup analysis or auxiliary analysis. If there are personnel reductions during the trial, indicate whether an intention-to-treat (ITT) analysis was used and compare it with per-protocol (PP) analysis]***

The primary and secondary indicators of this study's scales will be presented to participants in the form of paper questionnaires, while behavioral data and EEG data will be collected from participants using electroencephalography devices.

Demographic data and scale data analysis will use SPSS software, employing t-tests, covariance analysis, etc.; EEG data analysis will utilize Matlab_2013b software, EEGLab software, and scripts.

For power spectrum and functional connectivity analysis: EEG data will calculate the relative power of six EEG frequency bands at the single electrode and ROI levels (δ: 1-4Hz, θ: 4-8Hz, α: 8-13Hz, low β: 13-20Hz, high β: 20-30Hz, low γ: 30-48Hz). Phase Locking Value (PLV) and Phase Lag Index (PLI) will be calculated using the FieldTrip toolbox. Source localization analysis will be used to explore functional changes in subcortical regions.

For ERP data, we selected the N2 and P3 components based on previous research. The analysis epoch will be 1000ms, with the baseline being the 200ms before stimulus presentation. The N2 negative component is primarily induced by the frontal central region, so the frontal midline electrodes were chosen as the region of interest. The time window for N2 is 250-350ms post-stimulus lock, while P3 is a positive component appearing in the parietal mid/post region, with the parietal electrodes selected as the region of interest, and the time window is 300-400ms post-stimulus presentation. To avoid Type I error rates, we did not analyze individual electrodes; instead, we averaged the amplitude values of the electrodes within the selected time window as the dependent variable for analysis. All work related to ERP component analysis was conducted using ERPLAB.

Finally, we will simultaneously conduct ITT analysis for this study, considering dropout levels as a very important indicator for recording and analysis, attempting to objectively reveal the intervention effects of focused breathing meditation training on executive function from both ITT and PP analysis perspectives..

1. ***Participant management system.***

(1) Contact and Guidance: A contact group composed of relevant researchers is responsible for providing guidance or answers to all research-related content or questions encountered by participants throughout the duration of the study. This ensures that participants have a sufficient understanding of the research and can better cooperate with the process. Researchers must promptly address and record any instances of withdrawal, removal, loss to follow-up, termination, or suspension involving participants.

(2) Emergency Team: Comprised of researchers and psychological teachers from the university's mental health education center, this team will timely identify and intervene in cases of adverse reactions experienced by participants during the study. In situations that cannot be resolved, they will provide additional channels for addressing the issues faced by those participants. Every participant is encouraged and expected to proactively reach out to members of this team if they encounter any health-related problems at any time.

(3) Confidentiality of Information: Any information and data obtained about participants during the study will be kept strictly confidential. Information that could identify participants will not be disclosed to anyone outside the research team without the participant's consent. Any public reports related to the results of this research will not disclose the personal identities of the participants.

1. ***Specimen management system. [This includes the processes and systems for specimen collection, storage, and submission]***

This study does not involve the collection of samples.

1. ***Drug and equipment management system. [This includes the storage and distribution processes, checking systems, use, and recovery of drugs and equipment]***

This study does not involve the use of drugs or equipment management.

1. ***Data management system. [This includes data collection, management of source data and files, personnel responsible for data collection and entry, and checking systems]***

Before statistical processing, all collected data from participants will be uniformly anonymized, and measures such as data encryption or other secure storage methods will be implemented.

(1) Data Collection: Relevant data will be collected strictly in accordance with the standard operating procedures for different data collection methods.

(2) Management of Source Data and Files: Source data will be stored on the appropriate data platform, and multiple backups of the files will be created (such as storing them on a specialized, non-networked hard drive or an external hard drive used for data analysis). For some original data stored on third-party online platforms, timely downloads and secure storage will be conducted to ensure network security, enhance endpoint protection, prevent information leakage, maintain software and data backups, and regularly check for vulnerabilities and prevent virus intrusions.

(3) Personnel for Collection and Entry (Recording): The tasks will be handled by specially trained personnel.

(4) Verification System: Data quality will be improved through dual data verification and checks on the range of data values.

1. ***Composition and Responsibilities of the Data Security and Supervision Committee. [Describe the composition of the Data Security and Supervision Committee, including the members' names and contact information. Also, provide information on their roles and responsibilities in ensuring data security and monitoring the study]***

This study did not establish a separate Data Security and Supervision Committee.

1. ***Research Team. [Provide the names of the members of the research team. Describe their roles and responsibilities within the team]***

Project administration: CZF

Experimental design and data analysis: CZF, GQ, CZL, PXY, CLL

Psychological intervention and training: GJ

Recruitment of participants, data collection, and analysis: CZL, PXY, AXF, CQ

1. ***Intellectual Property. [Describe the ownership of intellectual property rights. Explain the policy and order for authorship attribution. Provide information on how the public can access and query the research plan]***

(1) Ownership of Intellectual Property: The sponsoring organization of the trial.

(2) Author Authorship Policy and Order: The order of authorship will be implemented according to the recommendations of the International Committee of Medical Journal Editors (ICMJE).

(3) Public Access to Research Plans: Information can be accessed through the published journals and the China Clinical Trial Registration Center along with the ResMan Clinical Trial Public Management Platform; the duration is 3-5 years.

1. ***Publication Plan. [Provide an estimated timeline and methods for publishing research reports. Specify the number of publications, including interim summary reports if applicable]***

(1) Expected Time and Method for Publishing Research Reports: The research report will be divided into several sections: the research plan section; analysis of panel data using various statistical methods; analysis of psychology-related data; and the comprehensive conclusion section.

(2) Expected Number of Publications: 1 article (excluding the publication of interim summary reports).

1. ***Plan for Sharing Raw Data. [Describe the plan for sharing raw data with the public. Specify the maximum time frame for sharing data, which should not exceed 6 months after the end of the trial. Explain the public platform that will be used for data sharing]***

The raw data will be uploaded to the ResMan Clinical Trial Public Management Platform, http://www.medresman.org.cn.

1. ***Treatment and Management of Participants after the Trial Ends. [Describe how participants who have not experienced improvement in their condition at the end of the trial will continue to receive medical care. Explain the measures that will be taken to ensure their ongoing treatment and management]***

Since the participants included in this study are all healthy university students and are guided by professional instructors in focused breathing meditation training, this training aims to explore the effects of a brief mindfulness intervention on executive function. Additionally, this process can help participants enhance their ability to self-regulate emotions when facing various stressors in real life. If, after the experiment, it is found that any participant's stress level reaches a clinically significant threshold, a psychological counselor from the university's psychological center will provide active intervention and, if necessary, refer them to nearby specialized hospitals for further support.

***References***

1. Center CINI. The 50th Statistical Report on the Development of Internet in China. 2022.

2. Center CINI. Annual Report on the Internet Use of Chinese Minors. 2021.

3. Ko CH, Király O, Demetrovics Z, Chang YM, Yen JY. Identifying individuals in need of help for their uncontrolled gaming: A narrative review of concerns and comments regarding gaming disorder diagnostic criteria. J Behav Addict. 2020;9(3):572-88. Epub 2020/10/05. doi: 10.1556/2006.2020.00058. PubMed PMID: 33011711; PubMed Central PMCID: PMCPmc8943683.

4. Li W, Garland EL, McGovern P, O'Brien JE, Tronnier C, Howard MO. Mindfulness-oriented recovery enhancement for internet gaming disorder in U.S. adults: A stage I randomized controlled trial. Psychol Addict Behav. 2017;31(4):393-402. Epub 2017/04/25. doi: 10.1037/adb0000269. PubMed PMID: 28437120; PubMed Central PMCID: PMCPmc5468481.

5. Zhou H, Liu H, Deng Y. Effects of short-term mindfulness-based training on executive function: Divergent but promising. Clinical Psychology and Psychotherapy. 2020;27(5):672-85. doi: 10.1002/cpp.2453.

6. Baer RA, Smith GT, Hopkins J, Krietemeyer J, Toney L. Using self-report assessment methods to explore facets of mindfulness. Assessment. 2006;13(1):27-45. Epub 2006/01/31. doi: 10.1177/1073191105283504. PubMed PMID: 16443717.

7. Yu S, Rodriguez MA, Deng Y, Xiao L, Liu X. The Toronto Mindfulness Scale: Psychometric Properties of the Chinese Version. Mindfulness. 2021;12(8):1976-84. doi: 10.1007/s12671-021-01655-z.

8. Wang M. Sex differences in internet gaming disorder in neural responses to gaming cues: Zhejiang Normal University; 2019.

9. Young KS. Internet addiction: The emergence of a new clinical disorder. CyberPsychology & Behavior. 1998;1(3):237-44. doi: 10.1089/cpb.1998.1.237.

10. Ko CH, Liu GC, Yen JY, Chen CY, Yen CF, Chen CS. Brain correlates of craving for online gaming under cue exposure in subjects with Internet gaming addiction and in remitted subjects. Addict Biol. 2013;18(3):559-69. Epub 2011/10/27. doi: 10.1111/j.1369-1600.2011.00405.x. PubMed PMID: 22026537.

11. Cox LS, Tiffany ST, Christen AG. Evaluation of the brief questionnaire of smoking urges (QSU-brief) in laboratory and clinical settings. Nicotine Tob Res. 2001;3(1):7-16. Epub 2001/03/22. doi: 10.1080/14622200020032051. PubMed PMID: 11260806.

12. Patton JH, Stanford MS, Barratt ES. Factor structure of the Barratt impulsiveness scale. J Clin Psychol. 1995;51(6):768-74. Epub 1995/11/01. doi: 10.1002/1097-4679(199511)51:6<768::aid-jclp2270510607>3.0.co;2-1. PubMed PMID: 8778124.

13. Huang C, Tang Y, Wang L, Xie D, Fan C, Gao W. Development of adolescent executive function scale. Chinese Journal of Behavioral Medicine and Brain Science. 2014;23(5):463-5. doi: 10.3760/cma.j.issn.1674-6554.2014.05.025.

14. Li J. Self-Rating Scale of Sleep(SRSS). China Journal of Health Psychology. 2012;20(12):1851.

15. Gong X, Xie X, Xu R, Luo Y. Psychometric Properties of the Chinese Versions of DASS-21 in Chinese College Students. Chinese Journal of Clinical Psychology 2010;18(4):443-6.

16. Watson D, Clark LA, Tellegen A. Development and validation of brief measures of positive and negative affect: the PANAS scales. J Pers Soc Psychol. 1988;54(6):1063-70. Epub 1988/06/01. doi: 10.1037//0022-3514.54.6.1063. PubMed PMID: 3397865.

17. Edlin JM, Leppanen ML, Fain RJ, Hackländer RP, Hanaver-Torrez SD, Lyle KB. On the use (and misuse?) of the Edinburgh Handedness Inventory. Brain Cogn. 2015;94:44-51. Epub 2015/02/07. doi: 10.1016/j.bandc.2015.01.003. PubMed PMID: 25656540.

18. Kroenke K, Spitzer RL, Williams JB. The PHQ-9: validity of a brief depression severity measure. J Gen Intern Med. 2001;16(9):606-13. Epub 2001/09/15. doi: 10.1046/j.1525-1497.2001.016009606.x. PubMed PMID: 11556941; PubMed Central PMCID: PMCPMC1495268.

19. Spitzer RL, Kroenke K, Williams JB, Löwe B. A brief measure for assessing generalized anxiety disorder: the GAD-7. Arch Intern Med. 2006;166(10):1092-7. Epub 2006/05/24. doi: 10.1001/archinte.166.10.1092. PubMed PMID: 16717171.

20. shan Q, li S. Diagnostic test of screening generalized anxiety disorders in general hospital psychological department with GAD-7. Chinese Mental Health Journal. 2015;29(12):939-44. doi: 10.3969/j.issn.1000-6729.2015.12.010.

21. Petry NM, Rehbein F, Gentile DA, Lemmens JS, Rumpf HJ, Mößle T, et al. An international consensus for assessing internet gaming disorder using the new DSM-5 approach. Addiction. 2014;109(9):1399-406. Epub 2014/01/25. doi: 10.1111/add.12457. PubMed PMID: 24456155.

22. Wang M, Dai X, Yao S. Development of the Chinese Big Five Personality Inventory(CBF-PI) Ⅲ:Psychometric Properties of CBF-PI Brief Version. Chinese Journal of Clinical Psychology. 2011;19(4):454-7.

23. Li T, Kato K. Measuring Adult Attachment： Chinese Adaptation of the ECR Scale. Acta Psychologica Sinica. 2006;38(3):399-406. PubMed PMID: CSCD:2455967.

24. Kong X, Hao Z. The Influence of Adult Attachment Style on Attitude Toward Professional Psychological Seeking Help:The Mediating Effect of the Expected Results of Self Disclosure. Chinese Journal of Clinical Psychology. 2021;29(5):1115-8. PubMed PMID: CSCD:7074077.

25. Farias M, Maraldi E, Wallenkampf KC, Lucchetti G. Adverse events in meditation practices and meditation-based therapies: a systematic review. Acta Psychiatrica Scandinavica. 2020;142(5):374-93. doi: 10.1111/acps.13225. PubMed PMID: WOS:000563911800001.
