# Supplementary material for: Protocol for a Randomized Controlled Trial to Enhance Executive Function via Brief Mindfulness Training in Individuals with Internet Gaming Disorder
Source: PLoS One. 2025 Apr 1;20(4):e0320305. doi: 10.1371/journal.pone.0320305 (PMC11960939; doi:10.1371/journal.pone.0320305)
Supplement: S3 File — (DOCX) [file pone.0320305.s003.docx]

| **S3 File. All items from the Chinese Clinical Trial Register (ChiCTR) Registration Data Set** | | | |
| --- | --- | --- | --- |
| **Registration number：** | ChiCTR2400081509 | **Registration Status：** | Prospective registration |
| **Date of Last Refreshed on：** | 2024/3/4 | **Date of Registration：** | 2024/3/4 |
| **Public title：** | The effect of brief mindfulness intervention on executive function in individuals with Internet Gaming Disorder | | |
| **Scientific title：** | The effect of brief mindfulness intervention on executive function in individuals with Internet Gaming Disorder | | |
| **Applicant's institution：** | Kunming University of Science and Technology | **Study leader：** | Zhuangfei Chen |
| **Approved by ethic committee：** | Yes | **Approved No. of ethic committee：** | KMUST-MEC-211 |
| **Name of the ethic committee：** | Medical Ethics Committee of Kunming University of Science and Technology | **Date of approved by ethic committee：** | 2023/10/30 |
| **Country：** | China | **Institution** | Kunming University of Science and Technology |
| **Source(s) of funding：** | Yunnan Fundamental Research Projects (202401AT070332) | **Target disease：** | Internet Gaming Disorder |
| **Study type：** | Interventional study | **Study phase：** | Active |
| **Study design：** | Parallel | **Study execute time：** | From 2024-03-05 to 2025-03-02 |
| **Objectives of Study：** | To explore whether the executive function of internet gaming disorder is damaged, to summarize the effect of brief mindfulness intervention on executive function and brain function mechanism, and to provide more targeted intervention strategies for the study of executive function. | | |
| **Inclusion criteria** | 1. Right-handed;  2. Having normal vision or corrected to normal vision;  3. Between 18 and 40 years old;  4. Normal and healthy mental and physical status (PHQ-9 total score < 20, GAD-7 total score < 11);  5.No other addictions (smoking, alcohol, gambling);  6. Internet gaming disorder :Online Game Addiction Scale score ≥ 50 ,DSM-5 score ≥ 5, game history (more than 2 years) and the game time (more than 14hours/week) Internet gaming entertainer:Online Game Addiction Scale< 50, DSM-5 score <5. | | |
| **Exclusion criteria：** | 1. Patients with asthma, contact dermatitis and allergy to silicone gel;  2. Those who have taken steroids in the past 3 months;  3. Practice any meditation practice or yoga, tai chi or qigong for more than 20 hours in the past year or lifetime, attend a meditation or yoga retreat, and attend any meditation course;  4. Not suitable for electroencephalography (such as metal implants and severe cranial injuries);  5. Total score of PHQ-9 ≥ 20; GAD-7 total score ≥ 11;  6. Have other addictions (smoking, alcohol, gambling)  7. Advocating specific religious beliefs, which makes it impossible to meditate according to the needs of the course;  8. Currently participating in similar or other neurophysiological trials. | | |
| **Interventions：** |  | | |
| **Group (Sample size)：** | experimental group (n=61) | control group (n=61) | |
| **Intervention：** | Brief mindfulness training | Relaxation training | |
| **Primary Outcomes：** | Trait-, State-mindfulness； Electroencephalographic (EEG) and behavioral data； Gaming craving-related metrics； Impulsivity； Executive function | **Measure time point of outcome：** | -T2 and -T1 (baseline), T0 (post intervention), T1-T5 (follow-up; i.e., 1-week, 1-mon, 4-mon, 6-mon, 1-year) |
| **Randomization Procedure (please state who generates the random number sequence and by what method)：** | The statistician will assign the experimental group and the control group in a 1:1 ratio according to the order of the participant sequence table. Randomization for this trial will be performed using the random sequence Generator station, https://www.random.org/. | | |
| **Blinding：** | This trial is a group therapy, and the participants are all students in the same school, so it is inevitable that participants who know each other or do not know each other will talk about the contents related to the trial training they received. Blinding the researchers is also challenging, because it is easy to get caught up in the experiment. Therefore, the double blind requirements of clinical trials cannot be achieved. | **Calculated Results after the Study Completed public access:** | Public |
| **IPD sharing; The way of sharing IPD” (include metadata and protocol, if use web-based public database, please provide the url)** | Yes; http://www.medresman.org.cn | **Data collection and Management (A standard data collection and management system include a CRF and an electronic data capture：** | Raw trial data will be uploaded using the ResMan database, a public platform for clinical research. |
