# Supplementary figures and images for: Protocol for a Randomized Controlled Trial to Enhance Executive Function via Brief Mindfulness Training in Individuals with Internet Gaming Disorder
Source: PLoS One. 2025 Apr 1;20(4):e0320305. doi: 10.1371/journal.pone.0320305 (PMC11960939; doi:10.1371/journal.pone.0320305)

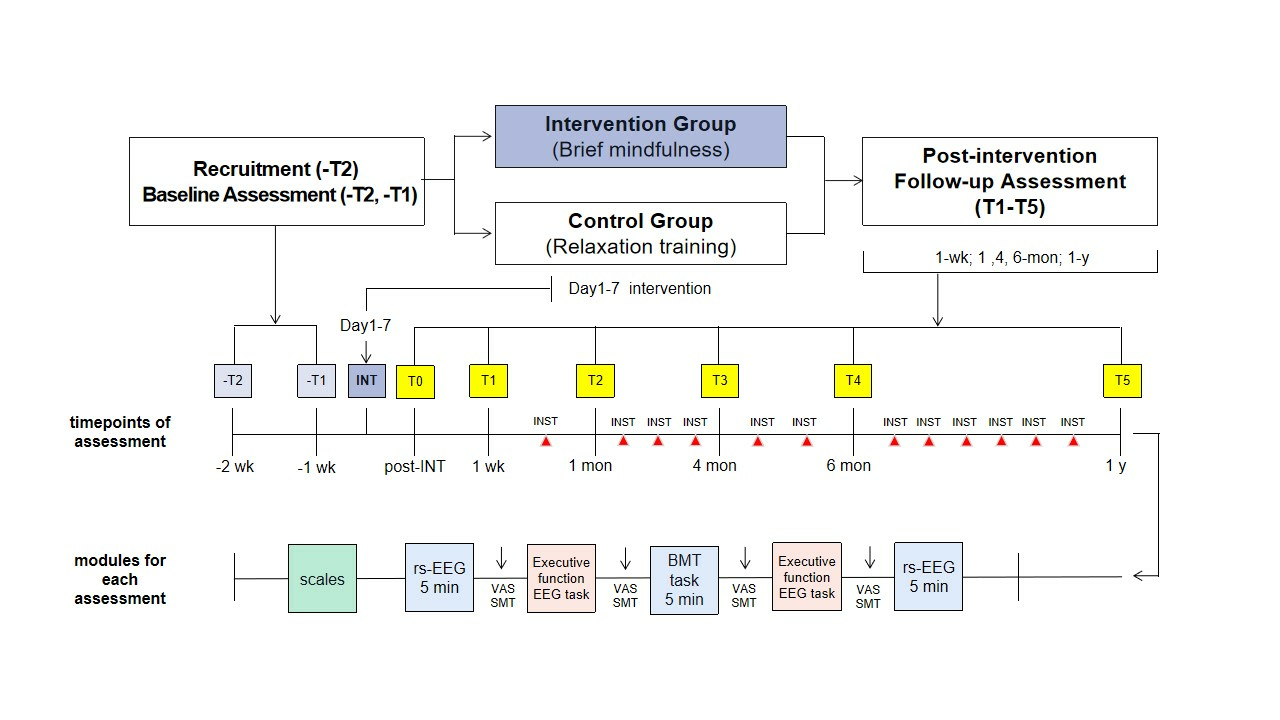

Supplement: Fig S1 — Note: The upper portion indicates the overall study design. The middle portion indicates the timeline for assessment of scales and EEG at baseline and post-intervention, also follow-up time-points. Brief mindfulness and relaxation training was administered to intervention group and control group after recruitment.Baseline assessment (-T2, -T1) was finished before intervention. Audio guidance was delivered for everyday’s practice for BMT group. There is no other practice and intervention for control group after training. The lower portion indicates modules of psychological and behavioral assessments during the baseline and 5 follow-up sessions. Executive function measurement was implemented via EEG tasks including Stroop, More-odd Shifting, N-back. INT: intervention, INST:monthly intensive training (red triangle); rs-EEG: resting-state EEG; BMT: brief mindfulness training; VAS: Visual analogue scale; SMT: state-mindfulness. (TIF) [file pone.0320305.s005.tif]
