# Supplementary material for: Protocol for a Randomized Controlled Trial to Enhance Executive Function via Brief Mindfulness Training in Individuals with Internet Gaming Disorder
Source: PLoS One. 2025 Apr 1;20(4):e0320305. doi: 10.1371/journal.pone.0320305 (PMC11960939; doi:10.1371/journal.pone.0320305)
Supplement: Attachment — Submitted filename: Response to Reviewers R1.docx [file pone.0320305.s007.docx]

Dear Editor and Reviewers,

We extend our sincere gratitude for the thoughtful feedback provided on our manuscript. We deeply appreciate the time and effort invested in reviewing our work. The insightful comments have been instrumental in significantly strengthening our research, leading to substantial improvements in both the methodology and presentation of our findings. The revised manuscript, thoughtfully incorporating all suggestions, is submitted for your kind consideration.

***Journal requirements:***

*When submitting your revision, we need you to address these additional requirements.*

*1. Please ensure that your manuscript meets PLOS ONE's style requirements, including those for file naming. The PLOS ONE style templates can be found at*

*https://journals.plos.org/plosone/s/file?id=wjVg/PLOSOne_formatting_sample_main_body.pdf and*

*<https://journals.plos.org/plosone/s/file?id=ba62/PLOSOne_formatting_sample_title_authors_affiliations.pdf>*

**Reply**

We have updated the manuscript and file names to comply with PLOS One's style requirements. Please see the resubmitted manuscript.

*Thank you for stating the following financial disclosure: [This study was supported by the National Natural Science Foundation of China (NSFC) (Nos. 32060196, 82360271 and 82201597), Yunnan Fundamental Research Projects (202401AT070332), Joint Funds for Medical Specialization of KUST (KUST-KH2023002Y), Yunnan Ten Thousand Talents Plan Young and Elite Talents Project (YNWR-QNBJ-2018-027, YNWR-QNBJ-2018-056) and The innovation team of stress and disorder in nervous system in Yunnan Province (202305AS350011).]. Please state what role the funders took in the study. If the funders had no role, please state: "The funders had no role in study design, data collection and analysis, decision to publish, or preparation of the manuscript." If this statement is not correct you must amend it as needed. Please include this amended Role*

**Reply**

The financial disclosure was updated to include all the NSFC grant numbers (Nos. 32060196, 32460211, 82360271 and 82201597). The funders had no role in study design, data collection and analysis, decision to publish, or preparation of the manuscript. The financial support was provided in forms of research materials. The authors have declared that no competing interests exist. The statement is included in the cover letter.

[Note: HTML markup is below. Please do not edit.]

***Review Comments to the Author***

***Reviewer #1:*** *Line 226: the randomization is unclear. State clearly how these 82 individuals with internet gaming addiction and 40 recreational gamers are allocated into the intervention and control group.*

**Reply**

Thank you for your valuable suggestions. Allocation will be obtained via email from staff uninvolved in other aspects of the trial. Participants were stratified by gender and IGD severity before random assignment to either the experimental or control group using a random number generator. This ensured a balanced distribution across groups. See Section 2.5, “Group allocation”, lines 358-362.

*Line 281: Several primary outcomes will be studied. Is the sample size calculated based on executive function sufficient to adequately support the analysis of the other primary outcomes?*

**Reply**

Thank you for your valuable feedback regarding the sample size calculation. Our study encompasses several primary outcomes, including scale scores, behavioral measures, EEG, and HRV. The sample size calculation prioritizes the effect size of executive function, particularly from Stroop task designs, as this represents the most challenging outcome in terms of both effect size and variance. This approach ensures sufficient statistical power to analyze all primary outcomes. A detailed explanation of this rationale is provided in lines 325-334.

*Line 292 for the progressive muscle relaxation training: To mention it will be held 7 days similarly with the intervention group.*

**Reply**

Thank you for your suggestion regarding the description of the progressive muscle relaxation training. We appreciate your attention to detail. To clarify, we will explicitly state in the manuscript that the progressive muscle relaxation training will be conducted over a period of 7 days, similar to the intervention group. We have modified lines 365-366 to include this information: “Simultaneously, participants in the control group will receive progressive muscle relaxation (PMR) training, which will be conducted over a period of 7 days, consistent with the intervention group.” Thank you again for your helpful feedback.

*Line 294: The sentence ‘following the intervention’ is to be omitted.*

**Reply**

Thank you for pointing this out; it has been corrected.

*Line 302: A separate subtitle to indicate intervention and control group respectively.*

**Reply**

We have revised the relevant section to include distinct subtitles for each group, ensuring that readers can easily differentiate between the intervention and control conditions (lines 380, 415).

*Information on the person who provide the training and do the data collection is to be provided.*

**Reply**

Thank you for your valuable feedback. We have clarified the trainers for the intervention and control groups, as well as the individuals responsible for data collection in each group (lines 381-385, 416-419).

*Page 22 Table 3: Information on whether all these inventories/questionnaires are in Chinese version and has been validated is to be provided.*

**Reply**

Thank you for your insightful feedback. We have added references to support the information regarding the Chinese version of the questionnaires, including their sources and validation status (line 487, and Table3).

*Data management on missing data if any is to be mentioned.*

**Reply**

Thank you for your valuable suggestion. We have revised the data management section regarding any missing data in lines 564-571. We will incorporate the assumption of missing at random (MAR) and use multiple imputation techniques to enhance accuracy. Additionally, we will conduct sensitivity analyses to evaluate the impact of different missing data scenarios.

*Line 476: Usually repeated measures ANOVA post hoc tests able to handle both within-group and between-group comparisons. Any reason why independent t test and paired t test are used?*

**Reply**

Thank you for your helpful comments. We have clarified that repeated measures ANOVA is best suited for analyzing data collected across multiple time points. The sentence at line 605 has been revised for clarity: "Repeated measures ANOVA will be applied to analyze data collected at different time points across multiple sessions."

*Line 478: The name of the effect size is to be provided.*

**Reply**

Thank you for your valuable suggestion. Cohen’s d and partial eta-squared values will be used to measure primary and secondary outcomes in order to assess treatment impact (lines 607-608).

*Line 478: Complete citation for SPSS 20.0 and R software including publisher name and version is to be provided.*

**Reply**

Revised, see lines 605-606.

*Line 478-479: Effect size with 95%CI to be stated.*

**Reply**

Revised, see line 610.

*The accepted statistically significant level is to be provided.*

**Reply**

Thank you for your feedback. We have added the statement: “Data yielding a p < 0.05 will be considered statistically significant”. Please see line 609.

*Line 496-499: To state clearly whether both intent-to-treat analysis and per protocol analysis will be conducted. If there are differs ‘n’ at various time points (due to attrition rates or missing data), using repeated measure ANOVA becomes problematic.*

*When performing repeated measures ANOVA with multiple time points, there is a risk of inflating the type I error rate (false positives) due to multiple comparisons. Thus, it is important to impose Bonferroni correction during post hoc testing to adjust the significance level for each comparison.
Figure 2: the analysis section requires revision.*

**Reply**

Thank you for the constructive suggestions. We will conduct both Intention-To-Treat (ITT) and Per Protocol (PP) analyses to assess the intervention effects. The ITT analysis preserves random assignment to reduce bias, while the PP analysis focuses on participants who completed the intervention. We acknowledge that variations in sample size at different time points may complicate repeated measures ANOVA. We will implement this analysis cautiously, accounting for sample size changes to maintain statistical power. Moreover, to prevent inflating the Type I error rate during multiple comparisons, we will apply Bonferroni correction in our post hoc testing to adjust significance levels, ensuring the reliability of our findings, (lines 632-645). The analysis section of Figure 2 has been revised based on the text content.

*References did not conform to the journal format.*

**Reply**

We are grateful for your valuable input. Each reference has been carefully reviewed and updated as necessary.

***Reviewer #2:*** *The manuscript presents a well-conceived study protocol that is likely to contribute valuable insights into the impact of mindfulness on executive function in individuals with IGD. By exploring the effects of mindfulness training on executive function in individuals with IGD, the authors aim to bridge a gap in current intervention methods and provide new therapeutic insights. The manuscript clearly defines both primary (e.g., EEG data, executive function) and secondary (e.g., anxiety, stress) outcomes, ensuring that both cognitive and psychological dimensions are covered. This comprehensive evaluation can provide a holistic understanding of the intervention's impact on IGD.
I only have some minor concerns for this well-documented manuscript.*

*1. Longitudinal Follow-up Justification: While the study’s longitudinal design is commendable, the rationale for the choice of follow-up intervals (e.g., one week, one month, etc.) could be strengthened. Are these intervals based on previous findings regarding the duration of mindfulness effects? Providing a clearer justification for these time points could clarify the expected temporal effects of mindfulness training.*

**Reply**

Thank you for your detailed and constructive feedback. We have clarified the rationale for our follow-up frequency selection:”This schedule is based on previous research showing that monthly follow-ups effectively monitor sustained benefits while balancing participant engagement and feasibility”. Supporting literature has been added. See lines 300-302.

2. *Control Condition (Progressive Muscle Relaxation): The choice of progressive muscle relaxation (PMR) as a control condition is appropriate for differentiating between general relaxation and mindfulness-specific effects. However, the manuscript could benefit from a brief discussion on whether PMR is expected to influence executive function or gaming cravings to any degree, as this could affect the interpretation of results.*

**Reply**

We are grateful for your suggestions. Accordingly, we have reviewed the relevant literature and provided additional clarifications.”Previous research observed slight gaming craving reduction with PMR, potentially due to a placebo effect. However, PMR group did not show significant changes in executive control network connectivity, and the difference from the mindfulness intervention group remained substantial. This highlights mindfulness' unique efficacy, as PMR uses a distinct relaxation mechanism”. See lines 422-427.

*3. Terminology and Consistency: Ensure consistency in the terminology, such as using “Internet Gaming Disorder” (IGD) consistently instead of alternating with "gaming addiction" to prevent ambiguity.*

**Reply**

Thank you for your detailed comments. We have carefully reviewed each inconsistent term and made the necessary revisions.

*4. Future Implications: The manuscript could be further strengthened by a more explicit discussion of how the findings may be translated into clinical practice, particularly for interventions beyond university-based or app-based contexts.*

**Reply**

Thank you for your valuable and insightful feedback. Based on your suggestions, we have supplemented the 'Strengths and Future Implications' subsection in the Discussion. Specifically, we have added content regarding ”The potential for brief mindfulness interventions could be integrated into various clinical settings, such as primary care clinics, community health centers, and mental health practices. For example, healthcare providers can incorporate brief mindfulness exercises into regular appointments, and community organizations can offer group sessions. Additionally, training programs for clinicians and educators can be developed to ensure they are equipped to deliver these interventions effectively. This approach would make the benefits of mindfulness more accessible to a broader population, including those with IGD, and support their executive function improvements in real-world settings”, see lines 684-692.

***Reviewer #3:*** *This manuscript presented an interesting RCT protocol that will examine the effectiveness of a brief mindfulness intervention on executive function in individuals that game (i.e., individuals with IGD and individuals without). I have provided comments on specific areas for improvement below.*

*Major issues:
1. Page 4, Rows 69-70: In the abstract, it may be beneficial to specify what you will be looking at in the EEG and what the behavioral metrics will specifically be measuring. Please also consider applying these changes to the main text of the manuscript (e.g., Page 21, Rows 370-371).*

**Reply**

Thank you for your valuable suggestions. We have explicitly included the EEG and behavioral measures to be assessed in the abstract (lines 72-75) and have also made corresponding additions in the main text (lines 474-480).

*2. Page 6, Row 113: I would recommend being slightly cautious of the phrasing here, as there is no consensus in the literature on the primary diagnostic characteristic of IGD. However, I agree that “excessive gaming behavior despite awareness of negative consequences” is one of the key characteristics of IGD.*

**Reply**

Thank you for your constructive suggestions. We have revised the description to: “The primary behavioral characteristic of IGD is excessive gaming behavior despite awareness of negative consequences...” (line 123).

*3. Page 7, Row 119: Given the focus on shifting and updating abilities throughout the manuscript, a brief description of each might be helpful to readers.*

**Reply**

Thank you for your valuable feedback. We have added a brief description of shifting and updating; see lines 117-122.

*4. Page 7, Rows 123: Before introducing the mindfulness sections, please provide more context on current IGD treatment by briefly discussing the most common intervention types for IGD, those that have shown promise, and any associated limitations reported in the literature. This may also provide more context for the section on Page 10, Row 193, that states issues around relapse in studies testing IGD interventions.*

**Reply**

Thank you for your valuable suggestion. We appreciate your guidance on providing more context regarding current IGD treatment approaches. We have included a brief overview of the most common intervention types and summarized their strengths and limitations. Please see lines 230-235 for details.

*5. Page 9, Rows 179-182: The discussion here appears to shift to smartphone addiction, which may overlap with but does not always encompass addictive gaming behaviors. Please consider making a clear link between smartphone addiction and IGD in this section.*

**Reply**

Thank you for your helpful suggestions. We have added necessary information on the relationship between PSU and IGD, which is summarized as followsz:“Earlier studies suggested that the severity of problematic smartphone use (PSU) exhibits similar traits to other forms of internet overuse, such as IGD. Additionally, the high accessibility and diverse range of smartphone applications may contribute to this overuse”,Please see lines 212-215 for details.

*6. Page 16, Row 314: Clarifying how, where, and when participants were encouraged to practice independently could strengthen this section.*

**Reply**

Thank you for pointing this out. Regarding the after-class exercises, we have added the following clarification:”The after-class exercises do not specify a time or location; they only need to be completed on the app on the same day.” Please see lines 388-389.

*7. Page 16, Row 333-337, and Page 17, Row 338-347: In the previous section 2.6, it states that the control group will be led by a psychology instructor. Could you please clarify the specific role of the psychology instructor, given the audio-guided nature of the progressive muscle relaxation training? If the instructor will not be providing the progressive muscle relaxation training, please clarify this in section 2.6 and perhaps state what exactly they will be responsible for (e.g., participant management).*

**Reply**

Thank you for your detailed review. We have supplemented the content to indicate that another psychology teacher oversees the control group and conducts audio-guided sessions. See line 416 and the last row of Table 2.

*8. Page 21, Rows 385-386: Please clarify what the data collection is referring to here, along with the total number of reminder messages that will be sent per timepoint if a participant does not complete evaluations/follow-up.*

**Reply**

Thank you for your feedback. We have added that we will send text message reminders to participants who have not completed data collection, especially for laboratory-based projects. See lines 495-496.

*9. Page 23, Row 397: This section mentions a pilot study of the current project. You could consider including some results and insights from the pilot study in the introduction section of the manuscript.*

**Reply**

Thank you for your valuable suggestions. Based on your feedback, we have provided the following elaboration on the pilot study and the corresponding paradigm parameter settings:”'The duration of stimulus presentation and the inter-stimulus interval will be determined based on the maximum response time of participants in the pilot study.” Additionally, “Executive Function Task 2 will be conducted without a practice session to reduce practice effects and prevent participant fatigue from prolonged task duration.” Please see lines 512-513 and 516-517.

*10. Page 27, Row 497: The manuscript raises an important point on dropout throughout the manuscript. If any strategies to reduce dropout have been planned, please discuss them in the manuscript. The following systematic review stated that the average follow-up retention rate was only 24% for internet-delivered interventions for internet-enabled addictive behaviors (inclusive of IGD) and discussed ways in which some studies addressed the risk of attrition: Park, J. J., King, D. L., Wilkinson-Meyers, L., & Rodda, S. N. (2022). Content and effectiveness of web-based treatments for online behavioral addictions: systematic review. JMIR mental health, 9(9), e36662.*

**Reply**

Thank you for your constructive suggestions. We have briefly summarized the methods incorporated into our study design to prevent participant dropout. Additionally, drawing on the review by Park (2022), we have outlined future research strategies. Please see lines 693-705 for more details.

*11. Table 2: You could consider providing a brief description of the following for readers unfamiliar with these practices or stories: (i) story of the old well and pebbles, (ii) raisin exercise, (iii) “life is now” reading, (iv) mountain meditation practice, (v) bean counting exercise.*

**Reply**

Thank you for your detailed suggestions. In light of your feedback, we have provided a brief summary of the five exercises in Table 2.

*12. Table 3: I am interested in knowing how “average daily gaming time” (under “primary outcome”) and “average weekly gaming time” (under “others”) differ in terms of how they will be measured and interpreted. The table states that participants will self-record their average daily gaming time – will this also be the case for average weekly gaming time?*

**Reply**

Thank you for your reminder. We have added the following note to Table 3:”b. Average weekly gaming time serves as an auxiliary criterion for determining addiction (as seen in the inclusion criteria); c. Average daily gaming time is used to explore the changes in gaming duration among gamers before and after the intervention (effect of mindfulness intervention)”.

*Minor issues:
1. Page 4, Row 57, and Page 6, Row 106: I recommend using “especially” instead of the shortened version.*

**Reply**

Revised accordingly.

*2. Page 4, Rows 58-59: Please indicate if this statement is specific to IGD or other disorders.*

**Reply**

Thank you for your feedback. We have revised the sentence to specify that it pertains to IGD, changing it to: “Mindfulness interventions have been indicated to improve executive functions to varying degrees in individuals with IGD” (line 60). This reflects that the study provided evidence of changes in symptom scores and activity in brain regions related to executive function, rather than direct improvements in executive function. Corresponding additions have been made in the main text (lines 197-204).

*3. Page 4, Row 60: Stating the three subcomponents in the abstract may improve clarity.*

**Reply**

Revised, see line 62.

*4. Page 4, Row 63: Since the acronym was introduced above, “Internet Gaming Disorder” could be omitted here.*

**Reply**

Revised, see line 65.

*5. Page 5, Row 80: Please add a space after the period. Similar issues have been noted in other areas (e.g., the period at the start of the paragraph on Page 12, Row 257, the repeated period on Page 14, Row 265, and a missing period in Table 2).*

**Reply**

Thank you for your feedback. We have individually revised each instance, including adding a space after the period on Page 5, Row 80, and addressing similar issues on Page 12, Row 257; Page 14, Row 265; and in Table 2.

*6. Page 6, Rows 95: Perhaps you could remove “which” here for better flow.*

**Reply**

Revised.

*7. Page 6, Row 115: I believe this should be written as “functional magnetic resonance imaging (fMRI).”*

**Reply**

Revised.

*8. Page 7, Row 120: Please clarify which part is controversial.*

**Reply**

Thank you for your valuable feedback. We have detailed the controversy regarding the impact of excessive gaming on cognitive flexibility, where mixed findings show potential neural enhancements but not in behavioral outcomes, leading to debate over its true effect. See lines 131-138 for more details.

*13. Page 9, Row 177: I would recommend changing “internet gaming addiction” to “internet gaming disorder” or “IGD” for consistency – please apply these changes to other sections of the manuscript that have used “internet gaming addiction.” Also, the reference (38) appears to not align with the statement it is supporting. I encourage you to double-check all in-text citations and the reference list.*

**Reply**

Thank you for highlighting this issue. We apologize for the inconsistency in our terminology. We have unified the terms related to IGD throughout the manuscript and have also carefully revised each reference to ensure consistency.

*14. Page 11, Rows 216-218: This sentence may be unnecessary in this section. If retained, it would be helpful if the sentence clearly reflects the primary and secondary aims stated in the abstract.*

**Reply**

Revised.

*15. Page 11, Row 220: As this is the “study design and participants” section, please briefly state the design of the trial. Please feel free to transfer a modified version of the sentence from Page 14, Row 284-285 here.*

**Reply**

Thank you for your helpful suggestions. We have added a brief statement of the trial design at the beginning of the 'Study Design and Participants' section. Please see lines 262-267 for details.

*16. Page 11, Rows 223-225: This sentence appears to be similar to the previous sentence. The two could be potentially combined to improve readability.*

**Reply**

Thank you for your valuable suggestions. We have merged two sentences to enhance readability. Please see lines 268-270.

*17. Page 11, Row 230: To maintain a formal tone, I would recommend removing “by the way.”*

**Reply**

Thank you for your valuable recommendations. We have replaced the informal phrase 'by the way' with the more formal 'moreover' to maintain a consistent tone. Please see line 286.

*18. Page 12, Row 239: Please put a space before the in-text citation.*

**Reply**

Revised.

*19. Page 15, Row 297: Please specify which behavior the brief mindfulness interventions have had moderate effects on executive function.*

**Reply**

Thank you for your feedback. We have added an explanation of the EF subcomponents, specifically the inhibition and updating subfunctions, at line 373.

*20. Page 15, Row 300: For consistency, I would recommend capitalizing the T in Figure 1.*

**Reply**

Thank you for your valuable suggestions. We have changed the 't' to uppercase in Figure 1 and its caption.

*21. Page 15, Row 304: It may be useful to specify if “school” refers to the university the study is recruiting from or multiple schools in a region.*

**Reply**

Thank you for your feedback. We have revised the text to explicitly state that the participants were recruited from the university (see line 382).

*22. Page 16, Row 313: Please specify if the post-session check-ins occur once a day or multiple times a day.*

**Reply**

We are grateful for your constructive suggestions. We have clarified that participants are required to check in at least once daily during the 7 consecutive days following each session, and the app automatically records practice duration during other times (lines 394-397).

*23. Page 17, Rows 353-354: I would recommend providing some examples of adverse events that may occur or directing the readers to section 2.12.*

**Reply**

Thank you for your valuable suggestions. We have provided specific examples of adverse events. Please see lines 449-452 for the details.

*24. Page 17, Rows 360-361: Please consider stating some examples of monitoring mechanisms.*

**Reply**

Thank you for your insightful feedback. We have provided specific examples illustrating the implementation of standardized procedures, personnel training, and monitoring mechanisms. Please see lines 460-464 for details.

*25. Page 24, Row 421, and Page 29, Row 538: I would recommend unbolding “This task will evaluate the updating component of executive” (Page 24, Row 421) and the letter “i” (Page 29, Row 538).*

**Reply**

Thank you for your detailed review. We have made the necessary revisions.

*26. Page 26, Row 474: Please clarify if those involved in data analysis will be blind to the intervention allocation.*

**Reply**

Thank you for your valuable suggestions. We have clarified that data analysts will be blinded to the intervention allocation to minimize bias during data analysis. Please see lines 600-601 for details.

*9. Page 37, Row 812: Please clarify if this is a thesis or peer-reviewed journal article.*

**Reply**

This is a master's thesis, and the relevant sections have been annotated. The reference is listed as entry 57, line 999.

*27. Table 1: In the main body of the manuscript, please state the reason for the chosen cut-off point for the Internet Addiction Test and cite supporting studies. You could also perhaps remove the fifth inclusion criterion, as it is the opposite of the sixth exclusion criterion. Lastly, please provide the full names of the acronyms provided in the table (e.g., GAD-7).*

**Reply**

Thank you for your feedback. We have supplemented the rationale for setting the IAT cut-off point by referencing previous studies of similar populations and using the Chinese version of the IAT questionnaire adapted from IGD; please see lines 272-278 in the main text. Additionally, we have removed the fifth inclusion criterion to avoid redundancy, and abbreviations have been appropriately annotated.

*10. Table 2: In the “post-class exercise (Ruixin app)” row, could you please clarify whether participants were instructed to listen to relaxation music? If applicable, please also indicate who provided this guidance and how it was communicated. Also, please remove the space after “meditation” in the Day 6 row.*

**Reply**

Thank you for your feedback. Based on your suggestions, we have made the necessary revisions. Post-class exercises are organized online by dedicated staff using the Ruixin app, which is capable of recording practice duration. This information has been added to Table 2.

*11. Table 3: Please consider changing “others” to “Others” for consistency.*

**Reply**

Revised.

*12. Figure 1: It may be helpful to provide an explanation of why primary and secondary outcomes were measured at both enrolment (-T2) and allocation (-T1).*

**Reply**

Thank you for your detailed comments. We have added the following clarification: “averages will be taken to reduce random errors.” This information can be found at line 368 and in the legend of Fig. 2.

*13. Figure 2: The caption for this figure could remove the acronyms for BMT, PMR, and CON, as they are not included in the figure.*

**Reply**

Revised.

*14. Figure 3: Please provide the unabbreviated term for HRV.*

**Reply**

Thank you for your feedback. We have added the information in the figure legend.

Thank you once more for your detailed review and constructive feedback. We are grateful for your valuable insights and look forward to any further recommendations you might offer to help us improve and refine our protocol.

Best regards,

Zhuangfei Chen

chen.zhf@outlook.com
